# Plates of the Cerebro-Spinal Nerves, with References; for the Use of Medical Students

**Published:** 1837-10

**Authors:** 


ART. VIII.—*Plates of the Cerebro-Spinal Nerves, with References; for the use of Medical Students.* By PAUL B. GODDARD, M.D., Prosector of Anatomy in the University of Pennsylvania, &c.—*Philadelphia,* 1837. 4to. pp. 60; 12 Plates.

THE present work is well adapted to answer the end for which it was published. The author does not profess originality, but acknowledges that his lithographs are culled from larger and more expensive works; amongst which those of Swan, Arnold, and Bell are most prominent. The copies, with the exception of some intentional modifications, are accurate; the references and numbering of the plates are also distinct, a matter of great moment in regard to the utility of plates of this sort; the facility of reference being not unfrequently sacrificed to the beauty of the engraving. Indeed, in any more expensive style of engraving than the present, it becomes essential to adopt the plan of Arnold and others, of giving the references on a separate page, with the plate in outline. The execution of some of the plates in the work before us is much better than that of others; but, on the whole, the volume is neatly got up, although, of course, far inferior to the quarto volume of Mr. Swan; the beauty and moderate price of which would scarcely make the publication of such a work as Dr. Goddard's a desideratum in England. It will, however, be highly useful to his own countrymen.

